# Classifying patients with non-specific chronic low back pain using the impact stratification score in an online convenience sample

**DOI:** 10.1186/s12891-023-06848-2

**Published:** 2023-09-09

**Authors:** Anthony Rodriguez, Patricia M. Herman, Mary E. Slaughter, Maria Orlando Edelen, Ron D. Hays

**Affiliations:** 1 RAND Corporation, Behavioral and Policy Sciences, 20 Park Plaza #920, Boston, MA 02116 USA; 2 RAND Corporation, Behavioral and Policy Sciences, 1776 Main Street, Santa Monica, CA USA; 3 RAND Corporation, Economics, Sociology, and Statistics, 4570 Fifth Ave #600, Pittsburgh, PA USA; 4 Patient Reported Outcomes, Value and Experience (PROVE) Center, Department of Surgery, Brigham and Women’s Hospital, Boston, MA USA; 5 Division of General Internal Medicine & Health Services Research, UCLA Departmentof Medicine, Los Angeles, CA USA

**Keywords:** Chronic low back pain, Impact stratification score, PROMIS®, Classification, Patient-reported outcomes

## Abstract

**Background:**

In 2014, the National Institute of Health Pain Consortium’s research task force (RTF) on research standards for chronic low back pain (CLBP) proposed the Impact Stratification Score (ISS) as a patient-reported outcome measure that could stratify patients by the impact CLBP has on their lives. This work compares three newly developed ISS-based classifications to the RTF’s original to provide an optimal recommendation.

**Methods:**

The online sample included 1226 individuals from Amazon’s Mechanical Turk who indicated having non-specific CLBP, average age of 40, 49% female, and 67% White. Participants completed the PROMIS-29 v2.1 profile survey that contains the 9 ISS items as well the Roland-Morris Disability Questionnaire (RMDQ) and Graded Chronic Pain Scale (GCPS). Other items included high-impact chronic pain; not working due to health problems; overall health; and number of healthcare visits for back pain in the past 6 months. Three new classifications were created using quartiles (Classification 2), latent profile analysis (Classification 3), and one modeled after the GCPS (Classification 4). Classifications were subsequently compared to the RTF-proposed classification (Classification 1) on several concurrent and prognostic criteria.

**Results:**

Classification 1 had three CLBP severity groups, four in Classification 2, three in Classification 3, and four in Classification 4. All novel classifications improved upon the original. Classification 2 performed best at minimizing the classification of those with negative outcomes into the lowest severity groups at baseline (e.g., 11% with RMDQ ≥ 7) and 6 months (e.g., 8.2% had fair/poor health). Classification 4 performed best at maximizing classification of those with negative outcomes into the most severe group concurrently (e.g., 100% had GCPS grade ≥ 2) and at 6 months (e.g., 100% with RMDQ ≥ 7).

**Conclusions:**

We developed three ISS-based classification schemes and tested them against several outcomes. All three improved upon the original scheme. While appearing more optimal than other classifications in the lowest severity groups, Classification 2 presents some considerations and limitations. Given that Classification 4 was an improvement at the lowest end of severity and was the best at the highest end, it is our tentative recommendation that this approach be adopted to classify individuals with non-specific CLBP.

**Supplementary Information:**

The online version contains supplementary material available at 10.1186/s12891-023-06848-2.

## Introduction

Non-specific chronic low back pain (CLBP) is a diagnosis of exclusion. Potential underlying pathologies (e.g., infection, tumor, fracture) are ruled out, leaving an otherwise heterogeneous pool of patients with only ongoing lumbar spine pain in common. Being able to subclassify this pool of patients into more homogeneous groups to better target treatment is the “Holy Grail” [1]   or “ultimate objective”[2] for CLBP research.

Classification schemes have been developed for many diseases–e.g., breast cancer, [3] hip or knee osteoarthritis, [4] heart failure, [5] and chronic and musculoskeletal pain [2, 6–9]— following several different prognosis research themes [10]. These schemes have been used in various ways to guide treatment and predict outcomes, but they also allow researchers to adjust for confounding, to design more efficient trials by reducing the heterogeneity of treatment effects, and to better compare results across studies [11]. For providers and patients, in addition to guiding treatment, classification could contribute directly to both diagnosis and prognosis.

All classification schemes have a goal of segmenting large diverse patient populations (e.g., patients with CLBP) into relatively homogeneous subgroups. However, homogeneity can be defined in at least three ways. First, subgroups could be similar in their current level of severity and concomitant effects. For example, subgroups with similar levels of chronic pain impact have similar healthcare costs, unemployment and absenteeism [12–15]. Second, subgroups could have similar future outcomes or recovery (i.e., prognosis), regardless of treatment [1, 2, 16]. Third, subgroup members could be similar in terms of their response to treatment targeted to their subgroup—i.e., members of each subgroup do better if they receive treatment designed for that subgroup [1, 2, 16]. Classification schemes that address these three types of homogeneity provide guidance for treatment. The first type (current severity) identifies those most in need of treatment. The second type (prognosis) identifies those who are more likely to respond to any treatment, and the third (targeted treatment) identifies the best treatments for each group. Note that one classification scheme may not result in all three types of homogeneity [1, 2].

In 2014 the National Institutes of Health Pain Consortium Research Task Force (RTF) on research standards for CLBP recommended that patients with CLBP be *stratified* by its impact on their lives [11]. In particular, the RTF felt that improved “prognostic stratification of patients with CLBP is important clinically to help guide the nature and intensity of therapy, and important for researchers to adjust for confounding and to improve comparability among studies” [11]^p2040^.

The Impact Stratification Score (ISS) was proposed as a measure of CLBP impact. It was defined as the sum of the raw scores of nine items from the Patient Reported Outcomes Measurement Information System (PROMIS) profile instrument, the PROMIS-29. The nine selected items cover physical function, pain interference, and pain intensity, resulting in a total score ranging from 8 (least impact) to 50 (greatest impact). Based on a sample of patients with LBP, with or without leg pain, who underwent epidural steroid injections, the RTF offered “relatively arbitrary” [11] ^p2037^ cutoff scores for classifying patients with CLBP: mild impact (ISS 8–27), moderate impact (ISS 28–34), and severe impact (ISS ≥ 35). Although the ISS has been evaluated as a continuous measure, [11, 17, 18] it has not yet been evaluated for stratification or classification.

In preparation for evaluating the ISS for use in classification, we recently published a scoping review of other published and studied classification schemes for CLBP that were based solely on patient self-reported measures [9]. The review identified five other schemes for the Subgroups of Targeted Treatment (STarT) back screening tool (SBST); [19] Multiaxial Assessment of Pain (MAP); [20] Graded Chronic Pain Scale (GCPS); [15] Back Pain Classification Scale (BPCS); [21–23] Chronic Pain Risk Score (CPRS) [24]. Four could be used to segment CLBP patients by current severity (SBST, MAP, GCPS, BPCS), all five to segment by prognosis, and one to target treatment (SBST). Each scheme was developed using a different method including clinical advisory panel review of statistically promising items and ROC curves; cluster analysis; Mokken analysis to develop a Guttman scale; stepwise discriminant analysis; and latent transition regression analysis. This study uses several of these methods to develop and test alternative CLBP classification schemes based on the ISS and then test them against cross-sectional and 3- and 6-month follow up data to identify the versions that are best at grouping individuals based on current severity and prognosis, respectively.

## Method

### Data Source and Design

In this observational study we used an online nonprobability convenience sample to collect data from individuals with non-specific CLBP using Amazon Mechanical Turk (MTurk) [25]. MTurk is a crowdsourcing marketplace or platform which pays temporary workers to complete discrete virtual tasks referred to as human intelligence tasks which include completing surveys, writing product descriptions, coding, or identifying content in images or videos. Baseline data were collected between August 21 and November 2, 2021, from 1972 high-quality, experienced MTurk workers (i.e., met the requester’s criteria for payment in ≥ 95% of tasks and previously completed 500 + tasks) who self-identified as having back pain on a general health survey. This survey included PROMIS® measures (e.g., global health, PROMIS-29), demographic items, and lists of health conditions, including whether they “currently have” back pain. Respondents completing this survey received $1.50.

Respondents who endorsed back pain were offered an additional $2.00 to complete a survey about their back pain. The back pain survey also included items about whether their back pain was due to a specific medical condition, what they did for pain management, and several back pain outcomes measures, including the 24-item Roland-Morris Disability Questionnaire, [26] the Graded Chronic Pain Scale, [15] and a single-item measure of high-impact chronic pain [27]. The survey included questions about whether the respondent’s back pain was chronic according to four definitions: 1) 3-month pain duration, 2) RTF definition, [11] 3) a health provider said you have it, and 4) you believe your back pain is chronic. Respondents to the back pain survey received follow up back pain surveys at 3 and 6 months.

For the present study, the original sample of 1,972 individuals who indicated having back pain was reduced to only those with CLBP based on any of the four definitions described above. This was further reduced to 1,230 respondents by determining whether CLBP was non-specific using a question that asked whether a healthcare provider told them their back pain was caused by a medical condition. From here 4 additional respondents were eliminated for incomplete data on the nine ISS items. The resulting final analytic sample (*N* = 1,226) includes individuals with the most common type of LBP, nonspecific LBP, [11, 28] who met at least one definition of chronic LBP, and completed all nine ISS items.

Baseline data were used to develop the CLBP classification schemes and to test for their ability to segment the population by severity. Three and six month follow up data were used to test the baseline classification schemes for their ability to segment the population by prognosis.

### Measures

We chose several outcomes by which to evaluate the success of each scheme. Most of the outcomes were used both at baseline to evaluate the success of the classification scheme in identifying groups with more homogeneous severity and at 3 and 6 months to evaluate success in identifying groups who were more homogeneous in terms of prognosis. These outcomes were also used as targets in the development of the classification schemes identified in the scoping review [9].

#### Primary Outcome for Severity and Prognosis

***Roland Morris Disability Questionnaire (RMDQ).*** The RMDQ is a 24-item measure assessing the impact of back pain on 24 daily activities and the scale score has a possible range of 0 (no disability) to 24 (maximum disability) [26]. Our primary outcome for both severity and prognosis was the proportion of individuals with CLBP who had a RMDQ score ≥ 7. Items from the RMDQ were also used as targets in the development of the Graded Chronic Pain Scale (GCPS), [15] and this specific cutoff was used in the development of the STarT Back Screening Tool [19].

#### Secondary Outcomes for Severity and Prognosis

***High-impact chronic pain.*** High-impact chronic pain was assessed using an item asking “Over the past 3 months, how often did pain limit your life or work activities?” [29] with responses options: 1 = never, 2 = some days, 3 = most days, 4 = every day. Responses of “most days” or “every day” indicated high-impact chronic pain and were coded as 1 with all else coded as 0.

***Overall health.*** General overall health was collected using an item from the PROMIS® Global Health survey [30]. Individuals were asked “In general, would you say your health is” with responses option from 1 = poor to 5 = excellent. Consistent with development of the revised GCPS, [27] responses of “fair” or “poor” were coded as 1 and all other responses coded as 0.

***Graded Chronic Pain Scale (GCPS)*.** The GCPS is a seven-item scale which has three pain intensity items and four disability items. The GCPS categorizes those with back pain into five disability categories from no pain problem to high disability: 0 = no pain, 1 = low disability/low intensity pain, 2 = low disability/high intensity pain, 3 = high disability/moderately limiting, and 4 = high disability/severely limiting [15]. Individuals who had a GCPS grade of 2, 3 or 4 [15] (used as the definition of clinically significant back pain in the Chronic Pain Risk Score [31]) were coded as 1 and those with a grade of 0 or 1 were coded as 0.

***Not working due to health problems*.** Participants were asked “What best describes your employment status?”. Response options included: Full time; Part time; Looking for work, unemployed, or temporarily laid off; Maternity/paternity leave; Not working due to health problems, permanent or temporary; Student; Retired; Keeping house or caring for a dependent. A binary variable was created such that individuals who indicated not working due to health problems were coded as 1, and all other responses coded as 0.

***5* + *healthcare visit for back pain.*** Respondents were asked about the number of times they had a healthcare visit for pack pain in the past 6 months. Individuals with 5 or more visits were coded as 1 and fewer than 5 coded as 0 (used as a prognostic indicator in the development of the GCPS) [15].

#### Impact Stratification Score (ISS)

The PROMIS-29 v. 2.1 instrument includes the 9 ISS items. Four items assess physical function regarding the ability to perform physical activities including walking, climbing stairs, chores around the house, and instrumental activities of daily living, such as running errands. Item responses range from 1 = without any difficulty to 5 = unable to do, with higher scores indicating poorer functioning. Four items assess pain interference with day-today activities, social activities, chores, and work around the home, with item responses ranging from 1 = not at all to 5 = very much, with higher scores indicating more pain interference. There is a single pain intensity item reflecting the intensity of pain a person experienced, on average over the past 7 days on a scale from 0 = no pain to 10 = worst pain imaginable, with higher scores indicating greater pain intensity.

### Analyses

This study uses updated versions of the methods used by three scoping study classification schemes that used empirically derived cutoff scores to identify subgroups. These schemes are the easiest to apply clinically as they only require upper and/or lower-bound cutoffs on simple total scores. We compare the newly developed versions of classification schemes based on the ISS to the “relatively arbitrary” 3-part scheme initially proposed by the RTF and rate them in terms of which identifies a set of classification groups that best differentiates individuals by severity and by prognostic value. Note that to test for the third type of homogeneity (identifying patients who are similar in terms of responding best to a treatment targeted to their group), a clinical trial would be required.

We compare the originally proposed 3-part RTF classification scheme [11] ^p2037^ (Classification 1) to three other ISS-based schemes. Classification 2 uses quartiles to categorize individuals into one of four groups. Classification 3 uses latent profile analysis (LPA) to identify heterogeneity within the sample and classify individuals based on patterns of profile-specific means. We fit models ranging from one to four profiles and examined fit statistics to determine if adding an additional profile improved model fit. To assess model fit, we used decreases in the negative two log likelihood (-2LL), Akaike Information Criteria (AIC), Bayesian Information Criteria (BIC), and the sample size adjusted Bayesian Information Criteria (aBIC). Further, we used non-significant Vuong-Lo-Mendell-Rubin Likelihood Ratio Test (VLMRT) and the Lo-Mendell-Rubin adjusted likelihood ratio test (LMRT) to evaluate if a *k*–1 profile solution (e.g., 4 vs 3 profile) is a better fit to the data. Models were estimated in Mplus v8.1. Classification 4 was modeled after the Graded Chronic Pain Scale [15]. Prior work [32] on the ISS has established the unidimensionality, monotonic nature of items, and hierarchical item difficulty comparable to the Mokken and Guttman scaling analyses conducted by the GCPS authors [15]. Consistent with the GCPS, this approach uses pain intensity to differentiate the least severe categories and relies on the ISS’s remaining eight items to classify the higher severity groups based on total sum scores. We compared performance of the four classifications schemes by examining associations with several outcomes at baseline and at 6-month follow-up as described above. For each classification we compared severity outcome prevalence in the least and most severe pain impact groups. The focus was on specificity–that is, the least severe pain impact group having the smallest percent of respondents with negative outcomes and the most severe group having the highest rates for negative outcomes, concurrently and prognostically. Analyses with 3 month follow up data were conducted and found consistent with 6-month results and are therefore not discussed but are presented in Supplemental Table S1.

## Results

Complete sample descriptive statistics for demographics and outcomes are presented in Table 1. At baseline, the mean age was 40; 50% were male 49% were female, and 1% were transgender or did not identify as female, male, or transgender. Sixty-seven percent were non-Hispanic White, 19% Hispanic, 7% non-Hispanic Black, and 7% non-Hispanic other race or multiracial. Seven percent reported a high school degree or less and 68% had a bachelor’s degree or higher. For the individual outcome measures, at all timepoints, missing data ranged from no missing to at most 1.9%.

**Table 1:** Demographics and characteristics of the final analytic sample

	M (SD) or *n* (%)
Age	40 (11)
Hispanic	231 (19%)
Race	
Non-Hispanic White	819 (67%)
Non-Hispanic Black	91 (7%)
Non-Hispanic Other race	61 (5%)
Non-Hispanic Multiracial	24 (2%)
Gender	
Female	600 (49%)
Male	617 (50%)
Transgender	4 (0.3%)
Other	5 (0.4%)
Education	
No high school diploma	2 (0.2%)
High school graduate or GED	83 (6.8%)
Some college, no degree	199 (16%)
Occupational/technical/vocational program	24 (2.0%)
Associate’s degree: academic program	81 (6.6%)
Bachelor's degree	628 (51%)
Master's degree (e.g., M.A., M.S., M.P.H., M.B.A.)	182 (15%)
Professional school degree (e.g., M.D., D.D.S., D.V.M., J.D.)	18 (1.5%)
Doctoral degree (e.g., Ph.D., Ed.D.)	6 (0.5%)
RMDQ ≥ 7	
Baseline	668 (55%)
6-months	172 (35%)
High-impact chronic pain	
Baseline	239 (20%)
6-months	68 (14%)
Bad/poor health	
Baseline	174 (14%)
6-months	123 (25%)
GCPS ≥ 2	
Baseline	625 (52%)
6-months	157 (32%)
Not working due to health problems	
Baseline	29 (2.4%)
6-months	21 (4.3%)
5 + healthcare visits for back pain (6 months only)	7 (9.2%)

*GED *General education development, *RMDQ* Roland-Morris Disability Questionnaire, *GCPS *Graded Chronic Pain Scale

### Classifications 1 and 2

Using the RTF classification based on ISS total sum score (Classification 1), respondents were first classified into three pain impact severity groups: mild (score 8–27; 80%, *n* = 985), moderate (score 28–34; 16%, *n* = 201), and severe (score ≥ 35; 3%, *n* = 40). Classification 2 used a quartile approach resulting in four pain impact severity groups: no impact (score 8–13; 25%, *n* = 310), mild (score 14–20; 29%, *n* = 351), moderate (score 21–26; 23%, *n* = 285), and high (score ≥ 27; 23%, *n* = 280).

### Classification 3

**Fig.1:**
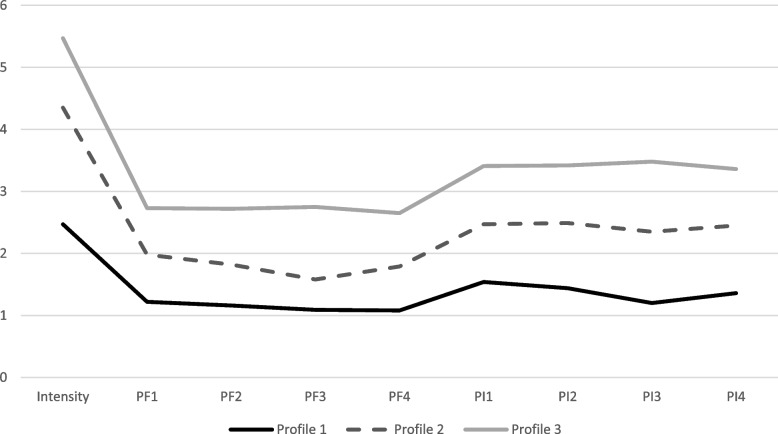
The three emergent pain severity groups from the latent profile analysis for the nine ISS items. Profile 1 (41%; *n* = 497) is characterized as no-to-low pain impact. Profile 2 (33%; *n* = 406) is characterized as mild pain impact. Profile 3 (26%; *n* = 323) reflects individuals with moderate-to-severe pain impact. PF = Physical function. PI = Pain interference

A series of LPA models were estimated and evaluated (see Table 2). Fit criteria identified the 3-profile model as the optimal solution such that all information criteria continued to decrease in size, and all likelihood ratio tests indicated that the 4-profile model was not better than the 3-profile model. Roughly, as can be seen in Fig. 1, the groups map onto no-to-low (Profile 1: 41%; *n* = 497), mild (Profile 2: 33%; *n* = 406), and moderate-to-severe (Profile 3: 26%; *n* = 323) pain impact categories.

**Table 2:** Model fit indices for substance use latent profile analysis

*Number of profile*	-2 LL	AIC	BIC	aBIC	VLMRT	*p*	LMRT	*p*
1 profile	32,930.84	32,966.838	33,058.846	33,001.670	-	-	-	-
2 profiles	28,390.78	28,446.777	28,589.899	28,500.959	4540.062	< 0.001	4477.106	< 0.001
**3 profiles**	**27,369.31**	**27,445.310**	**27,639.547**	**27,518.843**	**1021.467**	** < 0.001**	**1007.302**	** < 0.001**
4 profiles	26,844.44	26,940.444	27,185.796	27,033.328	524.866	0.0835	517.588	0.0859

### Classification 4

The four physical function and four pain interference items were summed to yield a total summary score ranging from 8 to 40. Three initial severity groups were first created based on these 8 items which corresponded to total sum scores ≤ 23 (i.e., average score less than 3 - aligning with average response options below “somewhat” and “with some difficulty”), scores between 24 and 31 (i.e., average score between 3 and 3.99 - aligning with average response options of “somewhat” and “with some difficulty”), and lastly scores ≥ 32 (i.e., average score of at least 4 - aligning with average response options of at least “quite a bit” and “with much difficulty”). The group with scores ≤ 23 was further stratified into two groups based on pain intensity. Inspection of cross-tabulated data for pain intensity and total sum scores for the remaining eight items showed clustering of higher intensity scores for the two most severe categories and greater variability in the lowest category. We therefore adopted a pain intensity split of < 5 (on a 0–10 scale) to further stratify the lowest pain impact group. That is, we split those with minimal interference and physical function impairment and *lower* intensity from those with similar interference and function but *higher* pain intensity. This classification resulted in four impact severity groups: low impact with low pain intensity (58%, *n* = 716), low impact with high pain intensity (28%, *n* = 339), moderate impact (13%, *n* = 161), and high impact (1%, *n* = 10).

### Association with outcomes

Across all classification schemes we compared performance on several dichotomized outcomes at baseline, 3 months (see Supplemental materials), and 6 months. Table 3 includes baseline results for the percentage of respondents with a negative outcome in the least and most severe pain impact groups across all classification schemes. Table 4 displays results at 6 months. Bolded values in tables reflect the best rates across classifications. For complete tables with all severity categories other than lowest and highest, see supplemental tables S2 for baseline results and S3 for 6-month results.

**Table 3:** Baseline outcomes analysis to identify the best* classification scheme for groups with similar severity

	Classification 1 *n* (%)	Classification 2 *n* (%)	Classification 3 *n* (%)	Classification 4 *n* (%)
RMDQ ≥ 7				
Lowest	448 (46%)	**35 (11%)**	97 (20%)	265 (37%)
Highest	37 (92%)	253 (92%)	294 (90%)	**9 (93%)**
High-impact chronic pain				
Lowest	109 (11%)	**4 (1.3%)**	11 (2.2%)	49 (6.9%)
Highest	30 (75%)	148 (54%)	157 (50%)	**10 (100%)**
Bad/poor health				
Lowest	124 (13%)	**17 (5.5%)**	36 (7.3%)	87 (12%)
Highest	12 (30%)	56 (20%)	59 (18%)	**5 (50%)**
GCPS 2,3,4				
Lowest	419 (43%)	**48 (16%)**	121 (25%)	221 (31%)
Highest	38 (95%)	238 (87%)	265 (84%)	**10 (100%)**
Not working due to health				
Lowest	14 (1.4%)	**1 (0.3%)**	2 (0.4%)	9 (1.3%)
Highest	7 (18%)	15 (5.4%)	17 (5.3%)	**3 (30%)**

*Lowest* reflects the category with the lowest level of pain impact severity and *highest* reflects the most severe pain impact category. Bolded values in tables reflect the best rates across classifications

*RMDQ* Roland-Morris Disability Questionnaire, *GCPS* Graded Chronic Pain Scale, *Classification 1 *RTF proposed classification, *Classification 2* Quartile approach, *Classification 3* Latent profile analysis approach, *Classification 4* Total sum score stratified by pain intensity

^*^Best is defined as the smallest number of individuals with each negative outcome being classified in the lowest severity category and largest number being classified in the highest severity category

**Table 4:** 6-month outcomes analysis to identify the best* classification scheme for groups with similar prognosis

	Classification 1 *n* (%)	Classification 2 *n* (%)	Classification 3 *n* (%)	Classification 4 *n* (%)
RMDQ ≥ 7				
Lowest	120 (28%)	**15 (8.2%)**	37 (13%)	83 (24%)
Highest	**12 (100%)**	57 (85%)	66 (86%)	**3 (100%)**
High-impact chronic pain				
Lowest	34 (8%)	**2 (1.1%)**	7 (2.5%)	19 (5.6%)
Highest	9 (75%)	35 (52%)	40 (52%)	**3 (100%)**
Bad/poor health				
Lowest	91 (21%)	**15 (8.2%)**	32 (12%)	61 (18%)
Highest	8 (67%)	35 (52%)	38 (49%)	**3 (100%)**
GCPS 2,3,4				
Lowest	112 (26%)	**15 (8.2%)**	40 (14%)	71 (21%)
Highest	**12 (100%)**	51 (76%)	58 (75%)	**3 (100%)**
Not working due to health				
Lowest	11 (2.6%)	**1 (0.5%)**	3 (1.1%)	7 (2.1%)
Highest	**4 (33%)**	11 (16%)	13 (17%)	**1 (33%)**
5 + HC visits for BP				
Lowest	4 (7.8%)	1 (10%)	**1 (5.6%)**	**2 (5.6%)**
Highest	0 (0%)	**3 (11%)**	3 (9.4%)	0 (0%**)**

*Lowest* reflects the category with the lowest level of pain impact severity and *highest* reflects the most severe pain impact category. Bolded values in tables reflect the best rates across classifications

*RMDQ* Roland-Morris Disability Questionnaire, *GCPS* Graded Chronic Pain Scale, *HC* Healthcare, *BP* =back pain, *Classification 1* RTF proposed classification, *Classification 2* Quartile approach, *Classification 3* Latent profile analysis approach, *Classification 4* Total sum score stratified by pain intensity

^*^Best is defined as the smallest number of individuals with each negative outcome being classified in the lowest severity category and largest number being classified in the highest severity category

#### RMDQ

At baseline, the percentage of respondents with RMDQ scores ≥ 7 in the lowest severity group was smallest (11%) in Classification 2 which indicates the best performance. In the most severe groups, the largest rate (93%) was found in Classifications 1 and 4, again reflecting the best performance. At 6-months, the percent of respondents with RMDQ scores ≥ 7 in the lowest severity groups was smallest (8%) in Classification 2. In the most severe groups, the largest rate (100%) was in Classifications 1 and 4. As can be seen in Tables 3 and 4, when comparing performance across the lowest severity groups at both baseline and follow-up, the three new classifications performed better than the RTF proposed classification with Classification 2 performing best. In the highest severity groups, Classification 4 outperformed the rest at both baseline and follow-up.

#### High-impact chronic pain

Baseline endorsement of pain limiting life or work, in the lowest severity groups, was smallest (1.3%) in Classification 2. In the most severe groups, the largest rate (100%) was found in Classifications 4. At 6 months, in the lowest severity groups, the rate was still lowest (1.1%) in Classification 2. In the most severe groups, the rate was highest (100%) in Classification 4. Like the RMDQ results, at both baseline and follow-up, performance in the lowest severity groups was better across all new classifications relative to the Classification 1, with Classification 2 performing the best. In the most severe impact groups, Classification 4 outperformed all other classifications at both baseline and follow-up.

#### Overall health

Respondent rates at baseline of fair or poor overall health in the lowest severity group were smallest (6%) in Classification 2. For those in the most severe groups, the rate of fair or poor health was largest (50%) in Classification 4. At 6 months, the percent of fair or poor overall health in the lowest severity group was smallest (8%) in Classification 2. For those in the most severe groups, fair or poor health was highest (100%) in Classification 4. Once again, baseline and follow-up performance in the lowest severity groups favored the newly developed classifications over the RTF method with Classification 2 performing best. In the most severe impact groups, Classification 4 did better than all other classifications at both baseline and follow-up.

#### GCPS Grade ≥ 2

In the lowest severity groups, the baseline rate of individuals being in GCPS Grades ≥ 2 was smallest (16%) in Classification 2. For the most severe groups, the highest rate (100%) was in Classification 4. At the 6-month follow-up, for the lowest severity groups, Classification 2 has the lowest (8%) rate. For the most severe groups, the rate was highest (100%) in Classifications 1 and 4. Performance across the lowest severity groups at both baseline and follow-up was better in the new classifications relative to the Classification 1, and once again favoring Classification 2. In the most severe impact groups, both baseline and follow-up results suggest that Classification 4 consistently had the highest rates.

#### Not working due to health problems

Baseline rates of not working due to health problems for respondents in lowest severity group were smallest (0.3%) in Classification 2. In the most severe groups, the highest rate (30%) was in Classification 4. At 6 months, rates in the lowest severity groups were smallest (1%) in Classification 2. In the most severe groups, the highest rate (33%) was found in Classifications 1 and 4. Consistent with all previous outcomes, performance at baseline and follow-up in the lowest severity groups was better in the new classifications over the RTF classification with a slight preference for Classification 2. For the most severe impact groups, Classification 4 had the highest rates at both baseline and follow-up.

#### Healthcare visits for back pain

At 6 months, the percent of respondents in the lowest impact groups with  5+ health care visits for BP was lowest (6%) in Classifications 3 and 4. In contrast with what was seen for other outcomes, Classification 4 emerged as one of the best performing classifications for the lowest severity groups. In the most severe pain groups, the rate was highest (11%) in Classification 2. Also, unlike previous outcomes, Classification 2 was the best among the most severe groups. That said, it is important to note, as can be seen in Table 4, rates across classifications were generally low and as such these results should be interpreted with that consideration.

## Discussion

This study investigated the performance of three newly developed classification schemes using the nine-item ISS, by comparing them to the RTF’s proposed classification of pain impact severity. The goal of this work was to improve the ability of the ISS to classify individuals with non-specific CLBP into homogeneous groups which would have the potential to clinically help guide the nature and intensity of therapy while also being valuable to researchers interested in improving the comparability of studies. Under both concurrent and prognostic criteria and with a focus on specificity (ensuring that the highest severity classification captured the largest number of those with negative outcomes and that the least severity classification captured the fewest), support was found in favor of all three newly developed classifications. That is, whether examining performance in the lowest or greatest impact severity groups, the original RTF proposed stratification was outperformed by all other classification schemes. Specifically, we found that across outcomes both concurrent and prognostic, Classification 2 (quartile approach) was best at minimizing the number of individuals with negative outcomes classified in the low severity groups. On the other hand, Classification 4 (total sum score approach), performed best at maximizing the number of individuals with negative outcomes who were classified in the high severity groups both concurrently and at 6 months, with one exception. Interestingly, Classification 3 (latent profile approach), while often better than the RTF’s proposed classification in the lowest severity group comparisons, was never the best.

While appearing more optimal than other classifications in the lowest severity groups, Classification 2 presents some considerations and limitations. Given that this approach is based on quartiles, there is not a clinically sound empirical basis for its cutoffs. These quartile cutoffs are sample dependent, and although they can be used for other studies, a different sample would have resulted in another set of cutoffs. Classification 2 performed better than Classification 1. However, since the cutoffs for Classification 1 were also likely to have been based on the creation of equal-sized groups (three in this case), the benefits of Classification 2 may be solely due to its creation of a smaller low severity group. The further segmentation of the low severity group deserves further exploration.

Ideally, a single classification would perform best at both extremes of severity; however, this was not the case in the current study. That said, all new classification schemes performed better than the RTF approach at the lowest end of pain impact severity and Classification 4 performed best in the most severe pain impact groups. Given that Classification 4 was an improvement at the lowest end of severity and was the best at the highest end, it is our tentative recommendation that this approach be adopted to classify individuals with non-specific CLBP. It is worth highlighting that this approach also has the distinct feature of separating out pain intensity to stratify the lowest severity groups based on observed patterns. Aside from offering an improved stratification, Classification 4 is straightforward and easy to implement in practice or research. Clinicians and researchers can simply sum the four physical function and four pain interference items and apply the thresholds described here. For respondents with total sum scores ≤ 23, pain intensity scores < 5 would then be used to further stratify the lowest pain impact groups.

This study had the benefit of a large dataset containing the PROMIS-29 v2.1 items as well as a number of useful outcomes necessary for evaluating the various classifications in individuals with non-specific CLBP, but also has limitations. The approach of using a sample of individuals with non-specific CLBP was purposeful as the ISS measure was proposed for use in patients with CLBP. However, these results may not generalize to other pain populations (e.g., patients with headache or hip pain). The sample also consisted of MTurk respondents who were predominantly non-Hispanic White and reported having, but not necessarily having been diagnosed with non-specific CLBP which may further limit the generalizability of these findings. Additionally, while most outcome criteria were valuable in the evaluation and comparison of classification performance, the 5 + health care visits for back pain did not emerge as a useful target, likely due to low endorsement. It is also important to note that the highest severity group in Classification 4 was very small relative to other classifications which may be due to this being a non-clinical sample. The study was also limited in that we only developed and tested three classification schemes against the original RTF scheme. For example, further splitting of the lowest group in Classification 4 may result in increased benefits such as was seen in going from the larger Classification 1 to smaller Classification 2 mild group cutoffs. Lastly, the focus of this work was on the initial development and testing of novel ISS classifications. That said, we acknowledge the need for future work to externally validate the proposed classification structures.

## Conclusion

The RTF suggested using the ISS for stratification and proposed a scheme. We developed three other ISS-based classification schemes and tested them against several outcomes. All three improved upon the original scheme. Our proposed scheme following the structure of the GCPS (Classification 4) may be the best of these. However, future work should consider whether additional refinements can be made to existing threshold to further improve precision of specificity as well as examine performance in a clinical sample of CLBP patients, ideally in the context of a randomized controlled trial.

### Supplementary Information

**Additional file 1:** **Table S1.** Complete Baseline Outcomes Analysis to Identify the Best* Classification Scheme for Groups with Similar Severity. **Table S2.** Complete 3-Month Outcomes Analysis to Identify the Best* Classification Scheme for Groups with Similar Prognosis. **Table S3.** Complete 6-Month Outcomes Analysis to Identify the Best* Classification Scheme for Groups with Similar Prognosis.

## Data Availability

The datasets generated and/or analyzed during the current study are not publicly available due to lack of participant consent to share their data but are available from the corresponding author on reasonable request.

## Abbreviations

aBIC: Sample size adjusted Bayesian Information Criteria
AIC: Akaike Information Criteria
BIC: Bayesian Information Criteria
CLBP: Chronic Low Back Pain
GCPS: Graded Chronic Pain Scale
ISS: Impact Stratification Score
LBP: Low Back Pain
LMRT: Lo-Mendell-Rubin Adjusted Likelihood Ratio Test
LPA: Latent Profile Analysis
MTurk: Amazon Mechanical Turk
PF: Physical Function
PI: Pain Interference
PROMIS: Patient-Reported Outcomes Measurement Information System
PROMIS-29: 29-Item PROMIS Profile Measure
RMDQ: Roland-Morris Disability Questionnaire
RTF: Research Task Force
VLMRT: Vuong-Lo-Mendell-Rubin Likelihood Ratio Test
