# Supplementary material for: Classifying patients with non-specific chronic low back pain using the impact stratification score in an online convenience sample
Source: BMC Musculoskelet Disord. 2023 Sep 9;24:719. doi: 10.1186/s12891-023-06848-2 (PMC10492344; doi:10.1186/s12891-023-06848-2)
Supplement: Supplementary file 1 — Additional file 1: Table S1. Complete Baseline Outcomes Analysis to Identify the Best* Classification Scheme for Groups with Similar Severity. Table S2. Complete 3-Month Outcomes Analysis to Identify the Best* Classification Scheme for Groups with Similar Prognosis. Table S3. Complete 6-Month Outcomes Analysis to Identify the Best* Classification Scheme for Groups with Similar Prognosis. [file 12891_2023_6848_MOESM1_ESM.docx]

Supplemental Materials

Table S1. Complete Baseline Outcomes Analysis to Identify the Best* Classification Scheme for Groups with Similar Severity.

|  | Classification 1 n (%) | Classification 2  n (%) | Classification 3  n (%) | Classification 4  n (%) |
| --- | --- | --- | --- | --- |
| RMDQ ≥ 7 |  |  |  |  |
| Category 1 | 448 (46%) | **35 (11%)** | 97 (20%) | 265 (37%) |
| Category 2 | 183 (93%) | 146 (46%) | 277 (69%) | 251 (74%) |
| Category 3 | 37 (92%) | 281 (78%) | 294 (90%) | 143 (91%) |
| Category 4 | **-** | 253 (92%) | - | **9 (93%)** |
| High-impact chronic pain |  |  |  |  |
| Category 1 | 109 (11%) | **4 (1.3%)** | 11 (2.2%) | 49 (6.9%) |
| Category 2 | 100 (51%) | 25 (7.2%**)** | 71 (18%) | 97 (29%) |
| Category 3 | 30 (75%) | 62 (22%**)** | 157 (50%) | 83 (53%) |
| Category 4 | - | 148 (54%) | - | **10 (100%)** |
| Bad/poor health |  |  |  |  |
| Category 1 | 124 (13%) | **17 (5.5%)** | 36 (7.3%) | 87 (12%) |
| Category 2 | 38 (19%) | 55 (16%) | 79 (19%) | 50 (15%) |
| Category 3 | 12 (30%) | 46 (16%) | 59 (18%) | 32 (20%) |
| Category 4 | - | 56 (20%) | - | **5 (50%)** |
| GCPS 2,3,4 |  |  |  |  |
| Category 1 | 419 (43%) | **48 (16%)** | 121 (25%) | 221 (31%) |
| Category 2 | 170 (87%) | 142 (41%) | 241 (60%) | 263 (79%) |
| Category 3 | 38 (95%) | 199 (71%) | 265 (84%) | 133 (85%) |
| Category 4 | - | 238 (87%) | - | **10 (100%)** |
| Not working due to pain |  |  |  |  |
| Category 1 | 14 (1.4%) | **1 (0.3%)** | 2 (0.4%) | 9 (1.3%) |
| Category 2 | 7 (3.5%) | 6 (1.7%) | 9 (2.2%) | 6 (1.8%) |
| Category 3 | 7 (18%) | 6 (2.1%) | 17 (5.3%) | 10 (6.2%) |
| Category 4 | - | 15 (5.4%) | - | **3 (30%)** |

*Best is defined as the smallest number of individuals with each negative outcome being classified in the lowest severity category and largest number being classified in the highest severity category.

*Lowest* reflects the category with the lowest level of pain impact severity and *highest* reflects the most severe pain impact category. Bolded values in tables reflect the best rates across classifications. Best is defined as the smallest value in the lowest severity category and largest value in the highest severity category. RMDQ = Roland-Morris Disability Questionnaire. GCPS = Graded Chronic Pain Scale. Classification 1 = RTF proposed classification. Classification 2 = Quartile approach. Classification 3 = Latent profile analysis approach. Classification 4 = Total sum score stratified by pain intensity.

Table S2. Complete 3-Month Outcomes Analysis to Identify the Best* Classification Scheme for Groups with Similar Prognosis

|  | Classification 1 n (%) | Classification 2  n (%) | Classification 3  n (%) | Classification 4  n (%) |
| --- | --- | --- | --- | --- |
| RMDQ ≥ 7 |  |  |  |  |
| Category 1 | 161 (30%) | **20 (9%)** | 51 (15%) | 100 (24%) |
| Category 2 | 59 (78%) | 79 (39%) | 99 (55%) | 80 (59%) |
| Category 3 | 20 (87%) | 59 (59%) | 90 (76%) | 53 (77%) |
| Category 4 |  | 82 (76%) |  | **7 (100%)** |
| High-impact chronic pain |  |  |  |  |
| Category 1 | 51 (9.5%) | **3 (1.3%)** | 8 (2.4%) | 27 (6.4%) |
| Category 2 | 31 (41%) | 19 (9.4%) | 36 (20%) | 31 (23%) |
| Category 3 | 18 (78%) | 24 (24%) | 56 (47%) | 35 (51%) |
| Category 4 |  | 54 (50%) |  | **7 (100%)** |
| Bad/poor health |  |  |  |  |
| Category 1 | 88 (16%) | **16 (7.2%)** | 32 (9.5%) | 65 (15%) |
| Category 2 | 25 (33%) | 41 (20%) | 52 (29%) | 30 (22%) |
| Category 3 | 10 (43%) | 30 (30%) | 39 (33%) | 24 (35%) |
| Category 4 |  | 36 (33%) |  | **4 (57%)** |
| GCPS 2,3,4 |  |  |  |  |
| Category 1 | 175 (33%) | **27 (12%)** | 63 (19%) | 108 (26%) |
| Category 2 | 61 (80%) | 79 (39%) | 100 (56%) | 89 (65%) |
| Category 3 | **22 (96%)** | 64 (64%) | 95 (81%) | 55 (80%) |
| Category 4 |  | 88 (81%) |  | **6 (86%)** |
| Not working due to pain |  |  |  |  |
| Category 1 | 11 (2.1%) | **0 (0%)** | 2 (0.6%) | 9 (2.1%) |
| Category 2 | 8 (11%) | 6 (3%) | 8 (4.5%) | 5 (3.7%) |
| Category 3 | 6 (26%) | 5 (5%) | 15 (13%) | 8 (12%) |
| Category 4 |  | 14 (13%) |  | **3 (43%)** |

*Best is defined as the smallest number of individuals with each negative outcome being classified in the lowest severity category and largest number being classified in the highest severity category.

*Lowest* reflects the category with the lowest level of pain impact severity and *highest* reflects the most severe pain impact category. Bolded values in tables reflect the best rates across classifications. Best is defined as the smallest value in the lowest severity category and largest value in the highest severity category. RMDQ = Roland-Morris Disability Questionnaire. GCPS = Graded Chronic Pain Scale. Classification 1 = RTF proposed classification. Classification 2 = Quartile approach. Classification 3 = Latent profile analysis approach. Classification 4 = Total sum score stratified by pain intensity.

Table S3. Complete 6-Month Outcomes Analysis to Identify the Best* Classification Scheme for Groups with Similar Prognosis

|  | Classification 1  n (%) | Classification 2  n (%) | Classification 3  n (%) | Classification 4  n (%) |
| --- | --- | --- | --- | --- |
| RMDQ ≥ 7 |  |  |  |  |
| Category 1 | 120 (28%) | **15 (8.2%)** | 37 (13%) | 83 (24%) |
| Category 2 | 40 (82%) | 58 (35%) | 69 (52%) | 52 (51%) |
| Category 3 | **12 (100%)** | 42 (58%) | 66 (86%) | 34 (79%) |
| Category 4 | **-** | 57 (85%) | - | **3 (100%)** |
| High-impact chronic pain |  |  |  |  |
| Category 1 | 34 (8%) | **2 (1.1%)** | 7 (2.5%) | 19 (5.6%) |
| Category 2 | 25 (51%) | 10 (6.1%) | 21 (16%) | 20 (20%) |
| Category 3 | 9 (75%) | 21 (29%) | 40 (52%) | 26 (60%) |
| Category 4 | - | 35 (52%) | - | **3 (100%)** |
| Bad/poor health |  |  |  |  |
| Category 1 | 91 (21%) | **15 (8.2%)** | 32 (12%) | 61 (18%) |
| Category 2 | 24 (49%) | 42 (25%) | 53 (40%) | 34 (33%) |
| Category 3 | 8 (67%) | 31 (42%) | 38 (49%) | 25 (58%) |
| Category 4 | - | 35 (52%) | - | **3 (100%)** |
| GCPS 2,3,4 |  |  |  |  |
| Category 1 | 112 (26%) | **15 (8.2%)** | 40 (14%) | 71 (21%) |
| Category 2 | 33 (67%) | 48 (29%) | 59 (45%) | 51 (50%) |
| Category 3 | **12 (100%)** | 43 (60%) | 58 (75%) | 32 (74%) |
| Category 4 | **-** | 51 (76%) | - | **3 (100%)** |
| Not working due to pain |  |  |  |  |
| Category 1 | 11 (2.6%) | **1 (0.5%)** | 3 (1.1%) | 7 (2.1%) |
| Category 2 | 6 (12%) | 4 (2.4%) | 5 (3.8%) | 3 (2.9%) |
| Category 3 | **4 (33%)** | 5 (6.8%) | 13 (17%) | 10 (23%) |
| Category 4 | **-** | 11 (16%) | - | **1 (33%)** |
| 5+ HC visits for BP |  |  |  |  |
| Category 1 | 4 (7.8%) | 1 (10%) | **1 (5.6%)** | **2 (5.6%)** |
| Category 2 | 3 (14%) | 0 (0%) | 3 (12%) | 3 (14%) |
| Category 3 | 0 (0%) | 3 (15%) | 3 (9.4%) | 2 (12%) |
| Category 4 | - | **3 (11%)** | - | 0 (0%**)** |

*Best is defined as the smallest number of individuals with each negative outcome being classified in the lowest severity category and largest number being classified in the highest severity category.

*Lowest* reflects the category with the lowest level of pain impact severity and *highest* reflects the most severe pain impact category. Bolded values in tables reflect the best rates across classifications. Best is defined as the smallest value in the lowest severity category and largest value in the highest severity category. RMDQ = Roland-Morris Disability Questionnaire. GCPS = Graded Chronic Pain Scale. Classification 1 = RTF proposed classification. Classification 2 = Quartile approach. Classification 3 = Latent profile analysis approach. Classification 4 = Total sum score stratified by pain intensity.
